# Influence of Thermal Pollution on the Physiological Conditions and Bioaccumulation of Metals, Metalloids, and Trace Metals in Whitefish (*Coregonus lavaretus* L.)

**DOI:** 10.3390/ijms21124343

**Published:** 2020-06-18

**Authors:** Natalia A. Gashkina, Tatyana I. Moiseenko

**Affiliations:** Laboratory of Evolutionary Biogeochemistry and Geoecology, Vernadsky Institute of Geochemistry and Analytical Chemistry, Russian Academy of Sciences, 19 Kosygin St., 119991 Moscow, Russia; moiseenko.ti@gmail.com

**Keywords:** subarctic lake, thermal pollution, metal bioaccumulation, fish metabolism

## Abstract

The Kola nuclear power plant, which discharges warm water into one of the bays of subarctic Lake Imandra, significantly changes fish habitats. The temperature gradient of the lake is between 2 and 8 °C, which makes it significantly different from the natural temperature of the lake water. The stenothermal cold-water native species (lake whitefish (*Coregonus lavaretus* L.)), living for more than 40 years under conditions of thermal pollution, has adapted to this stressor. Moreover, this population differs favorably from the population in the natural-temperature environment in terms of its physiological state. Firstly, the hemoglobin concentrations in the fish blood are in the range of the ecological optimum, and secondly, it has a higher somatic growth, as estimated by Fulton’s condition factor. One of its main adaptive mechanisms of ion regulation is an intense metabolism of Na due to the high respiratory activity of the whitefish in warmer water. An increased accumulation of Rb and excretion of Se, Mo, and Si are associated more or less with that feature. Under conditions of an increased water temperature, the main metabolic need is due to a deficiency of Se in fish. The intensive metabolism of selenoproteins may involve risks of toxic effects and the bioaccumulation of Hg, As, and Cu in cases of increased existing stressors or the appearance of new ones.

## 1. Introduction

Power plants that discharge heated water into basins significantly affect the coastal ecosystems of cooling ponds. The Kola nuclear power plant (KNPP), built in 1973, provides about 50% of the electricity to the Murmansk region. Passing through the cooling system, the wastewater enters the Molochnaya Bay of subarctic Lake Imandra, with an amount of 1218 million m^3^ per year [1]. A thermal pollution area of up to 25 km^2^ was formed in the lake, with a temperature gradient from 8 °C, near the mouth of the discharge channel, and a gradual decrease as the heat flux spreads [2].

In the framework of classical ecotoxicological models, population vulnerability is defined as the product of three components—external exposure, intrinsic sensitivity, and population sustainability [3]. The first depends on a species’ food choice and active avoidance; the second depends on many traits, including assimilation efficiency, toxicant elimination ability, toxicant sequestration, and biotransformation potential; and the third depends on several traits related to recolonization, recovery, and dispersal ability. The secondary impacts of climate warming [3,4] or thermal pollution [5] include changes in nutrient cycling, primary productivity, and feeding relationships in food webs. An ecosystem with new characteristics was formed in the thermal affected area of KNPP, in which an increased bio-productivity was noted, as a result of which a large food supply was provided for fish [2].

The most common cold-water native species in Lake Imandra is the lake whitefish (*Coregonus lavaretus* L.). Attracted by the large food resources in the thermal pollution area, the whitefish overcame its internal sensitivity to thermal stress and showed a local adaptation. Between 1978–1996, cases of hermaphroditism were recorded in the affected thermal area. A vividly described individual was a whitefish (length 35 cm, weight 550 g, age 7+), in which both gonads had an abnormality: the testicle occupied the head part, and the ovary was further, and again transformed into the testicle in the tail part [6]. The normal function of the hypothalamic–pituitary–gonadal system is modulated by a variety of external stimuli, such as food availability, photo-period, behavioral interactions, and, particularly in poikilotherms, such as fish, temperature [7]. Additionally, in the period of 1978–1996, the number of fish with symptoms of general intoxication, kidney pathologies, and gonadal abnormalities increased slightly [6]. However, the most sensitive and vulnerable system of the blood formation of fish showed adaptive effects: if in the late 1970s, there was a huge variation in the concentrations of hemoglobin in the blood from 44 to 163 g/L, leukocytosis, and pathological changes in red blood cells (both hemolysis and vacuolization), then by the 1990s, the hemoglobin concentrations in the blood varied at an ecological optimum (80–130 g/L), and the morphological pattern of the blood did not differ from the normal pattern [8]. For example, adapted to elevated temperatures, freshwater mussels (*Anodonta anatina*) from a cooling pond of a power station showed a higher thermotolerance of the hemolymph (the lowest levels of nuclear anomalies and a high index of lysosomal membrane stability), compared to mussels from a site with a normal temperature regime, in experiments with moderate (25 °C) and extreme (30 °C) warming [9].

The models of the global climate change predict a 5–8 °C increase in the mean air temperatures by the year 2100 in Northern and Central Europe [10]. The Arctic species are the most vulnerable to climate warming and its effects on wildlife and fish [11]. Thus, further climate warming can affect species in the natural environment and exacerbate the effects of the existing thermal pollution.

Whitefish living in the thermal pollution area are good subjects for studying the effects of long-term exposure to elevated temperatures (over 40 years). The aim of the study was to examine the physiological conditions and bioaccumulation of metals, metalloids, and trace metals in whitefish organs and tissues under thermal pollution, as well as to identify both positive effects and potentially vulnerable metabolic sites, based on the bioaccumulation and the physiological state of fish.

## 2. Results

### 2.1. Habitat Conditions

Preliminarily, it is worth comparing the water chemistry conditions of the whitefish habitats in the thermal affected area of KNPP (area I-7) and in the natural environment (area I-8). The conductivities of water were approximately 61.0 and 53.6 μS/cm, Ca^2+^—3.74 and 3.59 mg/L, Mg^2+^—1.12 and 1.09 mg/L, Na^+^—7.27 and 6.25 mg/L, K^+^—1.47 and 1.30 mg/L, HCO_3_^−^—311 and 295 μeq/L, SO_4_^2−^—10.6 and 8.80 mg/L, Cl^−^—2.62 and 2.23 mg/L, TP—6.5 and 5.5 μgP/L, TN—176 and 168 μgN/L, and organic matter—3.57 and 4.28 mg/L in areas I-7 and I-8, respectively. The water chemistry parameters are quite close, and the pH was around 7.14. The decrease in the concentration of the organic substances in I-7 water can probably be explained by the more intense destruction processes in warm water. The concentrations of essential and non-essential metals, metalloids, and trace metals are also quite close in the water from the two areas (Table 1 and Table 2). However, the Pb content is significantly higher in the water of area I-7, which is possibly due to the highway influence. A higher content of Br and lower concentrations of Fe, Al and Zn are observed in the water of area I-7, compared to those of I-8. Notably, the concentrations of toxic metals, metalloids, and trace metals did not exceed their maximum permissible concentrations, as established by the national guidelines for fishery water bodies [12].

### 2.2. Physiological Condition

**Figure 1:**
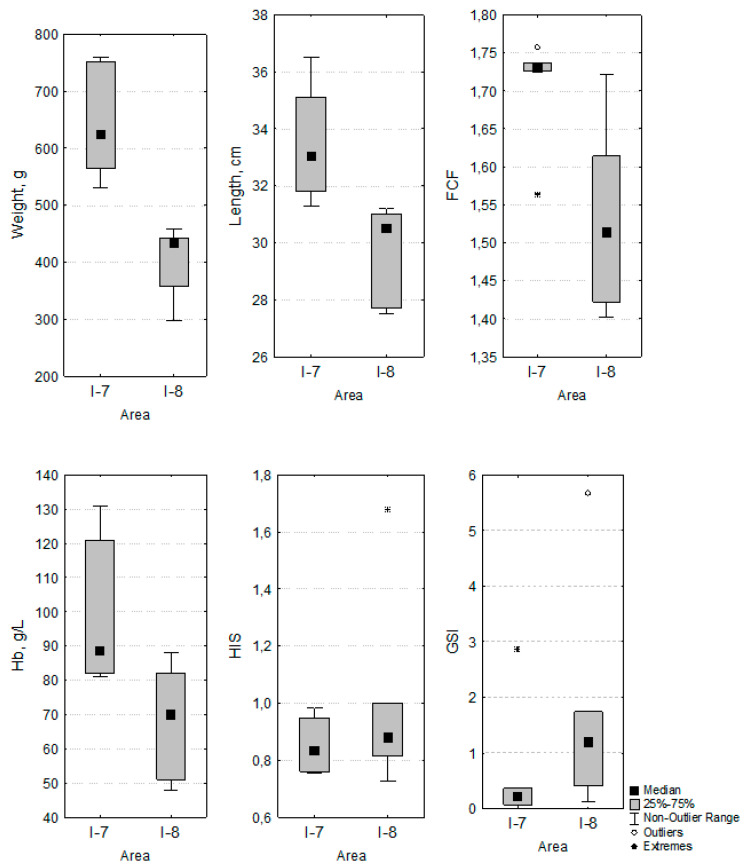
Weight, length, Fulton’s condition factor (FCF), blood hemoglobin concentration (Hb), hepatosomatic index (HIS), and gonadosomatic index (GSI) of the whitefish from the thermal pollution area (I-8) and the natural habitat area (I-8).

The physiological state of the fish was evaluated by the weight, length, FCF, blood hemoglobin concentration, HIS, and GSI. If the first four parameters were significantly different in fish from the two areas, the last two did not differ (Figure 1). It should be noted that the blood hemoglobin concentrations were in the range of the environmental optimum (80–130 g/L) in whitefish from area I-7, while the blood hemoglobin concentration in more than half of the studied fish from area I-8 was below the normal value. While the GSI did not significantly differ between whitefish from these areas, whitefish from area I-7 were at an earlier stage of maturation, which is quite natural in conditions of warmer water. It is also important to note that one whitefish had the maximum GSI (2.86), with the minimum FCF (1.56), of all whitefish in the thermal affected area, while one whitefish had the maximum GSI (5.67), with the maximum FCF (1.72), of all whitefish from area I-8. Thus, the individual physiological state, pathological deviations, and maturation stage introduced dispersion into the range of the bioaccumulation of metals, metalloids, and trace metals in whitefish organs and tissues, but did not affect the significance of the differences in the average values between the areas.

### 2.3. Bioaccumulation

The concentrations of such essential and conditionally essential metals and metalloids as Fe, Co, Se, Mo, B, Cr, and Li were significantly reduced in the gills of the whitefish from area I-7, compared to those from area I-8 (Table 1). Lower concentrations were observed for Mg, Na, P, Mn, Se, and B, but increased ones for Cu and Si, in the liver. Mg, Na, Se, Mo, Br, and Si accumulated less in the kidney. Only the S concentration was significantly higher in the muscles of the whitefish from area I-7, compared to those from area I-8. The accumulation of Ca, Na, and P was increased, and that of K, Se, Mo, B, Br, and Ni was decreased in the skeleton. Notably, the Se concentrations were low in almost all organs and tissues of the whitefish from the thermal affected area.

Among toxic metals, metalloids, and trace metals, only an increased accumulation of Rb and decreased accumulation of Be, Te, Sb, Al, Ti, Ba, and U were observed in the gills of the whitefish from area I-7 (Table 2). The accumulation of Hg, Zr and Rb was increased, but the accumulation of W, Sb and Al was reduced in the liver. The accumulation of Rb and As was greater, but that of Cd, W, Sb, Al, and Ba was lower in the kidney. An increased accumulation of Zr, Rb, and Cs was observed in the muscles of the whitefish from area I-7. The accumulation of As, Sr, Zr, and Rb was greater, while that of Hg, Sn, and U was lower in the skeleton. It should be noted that the accumulation of Rb was high in all organs and tissues of the whitefish from the thermal affected area.

## 3. Materials and Methods

The lake whitefish (*Coregonus lavaretus* L.) is the most widespread species. It is a bottom feeder that leads a relatively settled lifestyle, does not migrate over long distances and can be used as a good local bio-indicator of aquatic environments [6,13].

**Figure 2:**
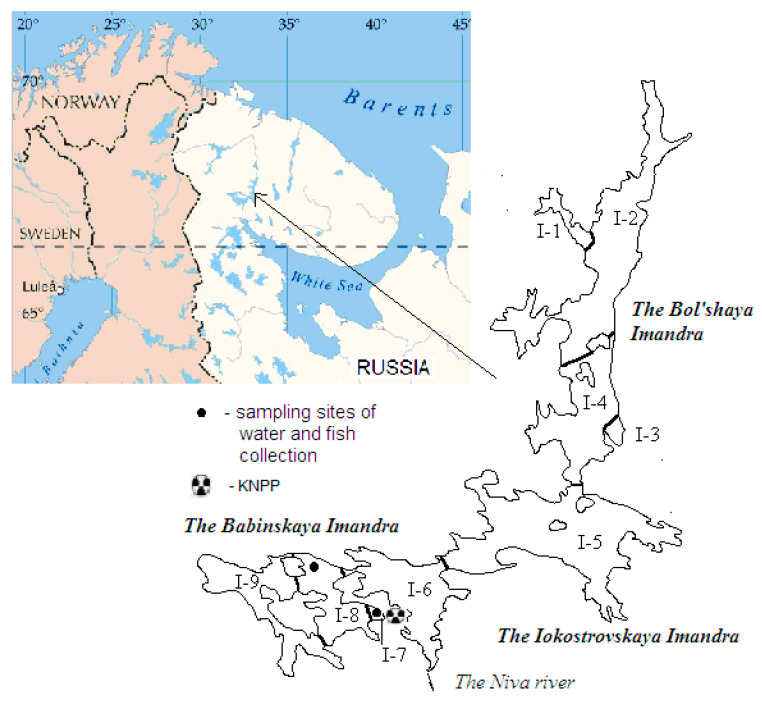
Map with the zoning of Lake Imandra.

The study was based on data obtained in 2018 on whitefish in the two areas of Lake Imandra (Figure 2). I-7 is the area of influence of warm water discharges from KNPP; and I-8 is the area not experiencing thermal influence, but the most similar in water chemistry. In this study, 12 fish (six from each area of the lake) were examined to determine the metal concentrations in their organs and tissues, as well as their physiological state.

### 3.1. Water Sample Collection

Water samples were taken from Lake Imandra at the exact sites where the fish were caught for examination (Figure 2). Water samples (two from each study area) were collected in Nalgene^®^ polyethylene bottles, immediately placed in dark containers, and then cooled to approximately 4 °C, while being transported to the laboratory. The Ca^2+^, Mg^2+^, K^+^, Na^+^, HCO_3_^−^, SO_4_^2−^, Cl^−^, total phosphorus (TP), and total nitrogen (TN) were analyzed in water using standard techniques. A Metrohm^®^ pH meter was used to measure the pH. The conductivity (20 °C) was measured with a Metrohm^®^ conductivity meter. The organic matter content was measured using the Mn oxidation method. The water samples were filtered using a Millipore system. The concentrations of metals, metalloids, and trace metals in the water samples were determined by ICP-AES (ICAP-61 Thermo Jarrell Ash, USA) and ICP-MS (X-7 ICP-MS Thermo Electron, USA) in the Institute of Problems of Microelectronics Technology and Superpure Materials RAS, Chernogolovka. The detection limits for the ICP-AES measurements were: Li: 0.5 mg/L; Si: 0.05 mg/L; Na, Mg, Al, Ca: 0.01 mg/L; K, Zn, S: 0.15 mg/L; P: 0.8 mg/L; Fe, Cu, Sr: 0.003 mg/L; and Mn, Ba: 0.0003 mg/L. The detection limits for the ICP-MS measurements were: Cd, Ba, Y, Zr, Nb, REE, Hf, Pb, Th, U: ≤ 0.1–1 ng/L; Ga, Ge, Rb, Sr, Sb: 1 ng/L; and Ti, V, Cr, Mn, Fe, Co, Ni, Cu, Zn, As: ≤10 ng/L. The detection limits for ICP-AES and ICP-MS were calculated as follows: DLi = Ci + 3α, where Ci is the mean content of an isotope of interest for measurements in control samples, and α is the standard deviation of its determination in control samples. For elements with several isotopes, DL was used for the most abundant isotope. The accuracy of the analyses was better than 10% for elements with C > 5 DL and did not exceed 20% for elements with C < 5 DL. For most of the analyzed elements, the concentrations were higher than 5 DL.

### 3.2. Concentrations of Metals, Metalloids, and Trace Metals in Fish Organs and Tissues

A special permit for fish catching was obtained for research purposes from the Murmansk Fisheries Committee. Fish were caught for use in the study. This was accompanied by simultaneous water sampling from the catch sites. To minimize seasonal and age-related changes, the fish were studied in the pre-spawning period (August–September), and fish of the same age group (4+–6+) were sampled. To analyze the metal concentrations, the gills, liver, kidney, muscles, and skeleton were sampled. Biological samples were dried to a constant weight at 105 °C. The dry samples were prepared for analysis by wet decomposition in concentrated nitric acid by adding hydrogen peroxide. The metal concentrations in the biological samples were determined by ICP-AES (ICAP-61 Thermo Jarrell Ash) and ICP-MS (X-7 ICP-MS Thermo Electron) in the Institute of Problems of Microelectronics Technology and Superpure Materials RAS, Chernogolovka. The detection limits for each element in the solutions of the decomposed biological samples were the same as in the analysis of the water samples. A certified reference material, DORM-3 and DORM-4 (Fish protein certified reference material for trace metals, National Research Council Canada, Canada), was also analyzed for quality control. The observed concentrations were within the certified standard ranges.

### 3.3. Physiological Condition

The hematology was analyzed using live fish immediately after catching them. The blood hemoglobin concentrations were determined using a portative hemometer (MiniGEM523, Russian Federation).

The following data were collected for each fish: body weight, length, and liver and gonads weight. From these measurements, Fulton’s condition factor (FCF) was calculated as follows [14]:

FCF = 100 × W/L^3^

where W is the total body weight of the fish (g), and L is the length (cm).

The hepatosomatic index (HIS) and gonadosomatic index (GSI) were calculated as follows:

HIS = 100 × (liver weight (g)/W), and GSI = 100 × (gonad weight (g)/W).

### 3.4. Statistical Analysis and Data Presentation

All statistical analyses were performed using Statistica. Independent samples were compared using *t*-tests to assess differences in the average metal concentrations in whitefish organs and tissues. All graphs were generated using SPSS, Statistica.

## 4. Discussion

Based on the application of bioaccumulation models that describe the uptake and distribution of chemicals in biotic systems with a temperature-dependent chemical fate and bioenergetics models, consumption and growth rates are predicted to be positively correlated with temperature in bioenergetics models but negatively correlated in bioaccumulation models [4]. In metabolic responses, it has generally been observed that the uptake and elimination of chemicals increase as temperature increases [7]. Indeed, reduced concentrations of both essential and non-essential metals, metalloids, and trace metals were observed in all organs and tissues, especially in the gills (Table 1 and Table 2). However, a number of metals, metalloids, and trace metals (Hg, Cu, As, Rb, Cs, and Zr) showed an increased accumulation, while a number of others (Zn, Tl, Pb, V, and Bi) did not show a temperature effect on bioaccumulation. For example, thermal pollution did not influence the bioaccumulation of Pb and As, unlike Cu, Zn, Hg and Cd, by concentrators, such as oysters, in a coastal area affected by the thermal water discharge of the Houshi Power Plant, China [15]. Due to the nature of an ion, its concentration in water can determine the permeation, metabolism and bioaccumulation of metals, metalloids, and trace metals, depending on the water temperature, but it can also be determined by the metabolic need of a species, as well as by many other external and internal factors [3,4].

### 4.1. Permeation, Metabolism, and Bioaccumulation of Metals, Metalloids, and Trace Metals Associated with Respiratory Activity and Ion Regulation

Fish homeostasis depends largely on the blood hemoglobin concentration. Therefore, to survive at elevated temperatures, individuals need to maintain a higher blood hemoglobin concentration, the optimal level of which determines the ability of an individual to survive. Whitefish with a blood hemoglobin concentration below normal can have a faster fatal outcome in the thermal pollution area, compared to fish surviving with anemia under normal temperature conditions (Figure 1). Moreover, the frequency of the respiratory cycle (operation of mouth and gill pumps) depends on the temperature. Thus, gill ventilation increases with an increasing water temperature. Additionally, such facts contribute to the acceleration of the respiratory rate, which involves, firstly, the oxygen saturation of water decreasing with an increasing temperature (the oxygen solubility in the water drops by almost 2 times from 0 to 30 °C), and secondly, the oxygen consumption increasing when several large amounts of organic matter are decomposed in the water column and detritus at the bottom in the thermal affected area.

Based on the blood hemoglobin concentration, the Fe absorbed by the gastrointestinal tract is quite enough, and the hematopoiesis is not limited. The Fe content is maintained in the kidney, although the Fe accumulation in the gills was much lower than that of the whitefish from area I-8 (Table 1). A reduced Al accumulation was noted in the gills, liver, and kidney of the whitefish from area I-7 (Table 2), which can be explained by the fact that Al appears to use Fe transport during its active metabolism. In humans, approximately 90% of plasma Al is associated with transferrin (the Fe^3+^-carrying glycoprotein in plasma) [16]. Another consequence of intensive erythropoiesis and blood circulation is probably the practically absent bioaccumulation of Sb (Table 2), which is negatively correlated with the blood hemoglobin concentration in the gills, liver and kidney (Table 3). More than 95% of Sb [III] in the blood was incorporated into red blood cells; on the other hand, about 90% of Sb [V] was found in the plasma; the excretion of Sb is rapid; and Sb [V] is mainly excreted in the urine and Sb [III] in the feces [17].

The high respiratory activity of whitefish in the thermal affected area is aimed at maintaining the efficiency of the gas exchange of O_2_ and CO_2_ with the water. While a high proportion of CO_2_ is eliminated by passive diffusion across the gill epithelia, carbonic anhydrase catalyzes the hydration of CO_2_, with the formation of H^+^ and HCO_3_^−^ ions in the cytosol of epithelial cells. To avoid metabolic acidosis, excess H^+^ should be eliminated. The following transport systems are located along the apical membrane: the first is the electroneutral Na^+^/H^+^–exchanger (NHE), which transports H^+^ across the apical membrane in exchange for Na^+^; the second is H^+^–ATPase (VHA), which transports H^+^ out across the apical membrane, creating an electrochemical gradient for Na^+^ to diffuse across the apical membrane through the Na^+^–channel that may also be permeable to K^+^; and other electrolytes, such as Cu^+^ and Ag^+^. On the other hand, along the basolateral membrane, VHA, Na^+^/K^+^–ATPase (NKA), and the Na^+^/HCO_3_^−^–cotransporter (NBC) help to maintain a very low Na concentration in the cytosol and also removes HCO_3_^−^ from cells [18]. It should also be noted that fish need to be hyper-regulators in hypotonic fresh water (osmoregulation requirement) and maintain higher concentrations of Na and Cl in the blood than in water. Thus, in addition to the diffusion of Na^+^ through the Na^+^–channel, whitefish have intensive Na^+^ pumping in exchange for H^+^ due to their greater respiratory activity. While unreliable, the Na accumulation was slightly increased in the gills of the whitefish from area I-7, compared to those from area I-8 (Table 1). At the same time, whitefish were induced to eliminate excess Na under the thermal impact (Table 1), and the decrease in the Na concentration was enhanced in the kidney with an increasing blood hemoglobin concentration (Table 3).

The K^+^–channel KC, which moves K^+^ back across the basolateral membrane to the extracellular fluids, is structured so that it is also somewhat permeable to larger alkali metal ions, such as Rb^+^ and Cs^+^, but relatively impermeable to Na^+^ and Li^+^ [18]. The permeation and accumulation of Rb in all organs and tissues are significant for the whitefish from area I-7, unlike those from area I-8, whereas an increased accumulation Cs is observed only in the muscles (Table 2). Rubidium is the closest K congener, replacing it in all known biochemical processes. It is catalyzed by Na^+^/K^+^–ATPase, the K^+^/Na^+^/2Cl^−^–co-transporter, and K^+^–channels [19]. For example, a 10-fold increase in Rb and Cs bioaccumulation was observed in the muscles of whitefish (*Coregonus lavaretus*) from two volcanic lakes, unlike the whitefish from an artificial lake in Central Italy, and the K accumulation was also slightly increased, but the Na accumulation was slightly reduced [20]. The substitution of Rb^+^ for K^+^ can affect NKA, which maintains the concentration gradients of Na^+^ across both the apical and basolateral membranes, thereby enhancing the Na^+^ outflow. The accumulation of Rb in all organs and tissues of the whitefish from area I-7 was greater than that of the whitefish from area I-8 (Table 2). Moreover, the Rb accumulation in the gills, kidneys and muscles was enhanced with an increasing blood hemoglobin concentration (Table 3). In the kidney, the Rb accumulation appears to increase the efflux of Na, which was enhanced with an increasing blood hemoglobin concentration, namely, in the whitefish from the thermal affected area (Table 3).

The accumulation of Cu and Tl in the kidneys of the fish was increased with an increasing blood hemoglobin concentration (Table 3), which was associated more or less with K metabolism. A relationship between the blood hemoglobin concentration and Cu accumulation was found in the kidney, and not in the liver (the main organ of Cu storage). The Cu accumulation reached its maximum values in the liver of the whitefish from area I-7, and they were 2 times higher than those of the whitefish from area I-8 (Table 1). While the relationship between Cu and K transport is not clear, the Cu transporter, CTR1, in humans stimulates an increase of K in the cell plasma membrane [21]. For example, the redistribution of essential metals in the liver of breams (*Abramis brama* L.) revealed that the accumulation of Cu and K increased at the stage of anemia development, while it decreased at the stage of protective function mobilization and enhanced hemopoiesis, probably as a result of the depletion of Cu storage in ceruloplasmin and metallothionein [22]. Copper accumulation in the liver of the whitefish from Lake Imandra seems to be regulated by the hemopoiesis intensity and by the various intensities of the detoxification processes [23]. The bioaccumulation of Cu in the organs and tissues of the whitefish from area I-7 generally corresponds to tissue-specific patterns, but apparently reflects more closely the high permeation of Cu in the ultra-fresh soft water of the lake and especially under the influence of warm water, which enhances ion regulation and metabolism. For example, Cu^2+^ may be reduced to Cu^+^ by a Cu reductase on the gill surface, prior to uptake [24].

To avoid metabolic alkalosis, excess H^+^ should also be eliminated. The apical membranes possess a Cl^−^/HCO_3_^−^–exchanger or anion exchanger (AE), whereas the basolateral membranes possess a Cl^−^–channel and NBC [18]. Thus, an intensive uptake of Cl should also occur in whitefish from the thermal affected area, and Br, having an affinity for AE, must also permeate intensively into the fish organism. While the Br concentration in the water of area I-7 was about 1.5 times higher than that in the water of area I-8, Br is excreted intensively and not deposited in the skeleton of whitefish from the thermal affected area (Table 1).

An uptake of Ca^2^^+^ across the gill apical membrane can occur by diffusion through a selective epithelial Ca^2+^–channel (ECaC), and Ca^2+^ transport across the basolateral membrane occurs by the combined action of the plasma membrane Ca^2+^–ATPase (PMCA) and an Na^+^/Ca^2+^–exchanger (NCX) [18]. The intracellular free Ca is much lower (0.1–1 mM) than the extracellular free Ca [25]. However, there are very efficient homeostatic mechanisms to move Ca^2+^ out of the dissolved form and into other forms, such as organic molecules or particulates, like CaCO_3_ or Ca_3_(PO_4_)_2_, which can be stored in bones [18]. Because intracellular Ca is kept at a very low level, a gradient is formed on the apical membrane of the gills. Evidently, in addition to Ca uptake through the gills, Ca absorption from food through the epithelia of the fish gastrointestinal system may be greater in whitefish under the influence of warm water. A large mineralization of the skeleton and large accumulation of Ca and P in the skeleton of whitefish from area I-7 can indicate this fact (Table 1). Strontium, as a Ca homologue, was also deposited more in the whitefish skeleton under warm conditions than in the natural thermal environment, and the Sr accumulation increased in the gills and skeleton of whitefish, which were forced to maintain a high blood hemoglobin concentration (Table 3).

Divalent metals can also be transported by the ECaC across the apical membrane, and it appears that the affinities of the Ca^2+^–channels with Cd^2+^ and Zn^2+^ in fish are greater than those with Ca^2+^ [18]. However, the Zn concentrations are maintained at physiological levels in all organs and tissues of the whitefish from area I-7 (Table 1), and Cd is intensively excreted from the kidney (Table 2).

Magnesium is a cofactor for enzymes that transfer phosphate groups, such as the ATPases, involved in energizing the pumps for H^+^ and Ca^2+^ and the exchanger for Na^+^ and K^+^, and most cellular Mg is associated with ATP. As a result, Mg deficiency has been implicated in imbalances of Ca, Na, and K, including shifts in the K:Na ratio in fish [18]. A decrease in Mg concentration was observed in whitefish liver under the influence of warm water (Table 1). The relationship of Mg and P (r = 0.963, *p* < 0.001) in the liver may indicate a decrease in glycolytic capacity. Moreover, the P concentration in the fish liver decreases with an increasing blood hemoglobin concentration (Table 3). A decreased liver energy potential of whitefish from area I-7 can also be indicated by the relationship between P and Mn (r = 0.679, *p* < 0.01) and their lower concentrations in the liver of fish (Table 1). The toxic effects of Cu can influence energy metabolism. A reduction in glycolytic capacity may be dependent on a direct competition between Cu^2+^ and Mg^2+^ for protein-binding sites that will induce enzyme conformational changes, altering activity [26]. Freshwater fish absorb most of the required Mg^2+^ from their diet across the intestinal epithelia, and Mg^2+^ requires a transporter protein with a large binding site and a dehydration mechanism [18]. A decrease in Mg accumulation was also observed in the kidney of the fish from area I-7 (Table 1), i.e., renal excretion exceeds the re-absorption of Mg.

### 4.2. Metabolism and Bioaccumulation of Metals, Metalloids, and Trace Metals Associated with Somatic Growth

An analytical review of the biological effects of wildlife and fish in the Arctic [11] offers the following examples: The long-term warming of freshwaters is likely to alter fish growth rates, and cold-water native species, such as Arctic char and lake trout, grow less efficiently in warmer waters. A multi-year study of Arctic char revealed that fish were under greater metabolic stress and had severe glycogen depletion near the end of an abnormally warm summer, compared to colder years [27]. These examples show an acclimatization response to climatic variations. On the other hand, the whitefish from area I-7 adapted to temperature stress, spending energy reserves not only to maintain basic biological functions, but also to induce somatic growth. The FCF of the whitefish from area I-7 is significantly higher than that of the whitefish from area I-8 (Figure 1). The approximate average growth rate was 128 g/year for the whitefish from area I-7 versus 81 g/year for the whitefish from area I-8, whereas the main metabolic need is Se (Table 1).

**Figure 3:**
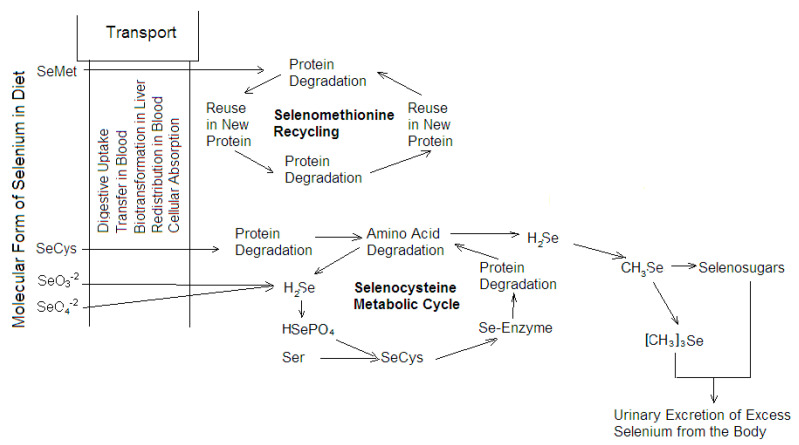
Selenium metabolism. Modified from Ralston et al. [35].

**Figure 4:**
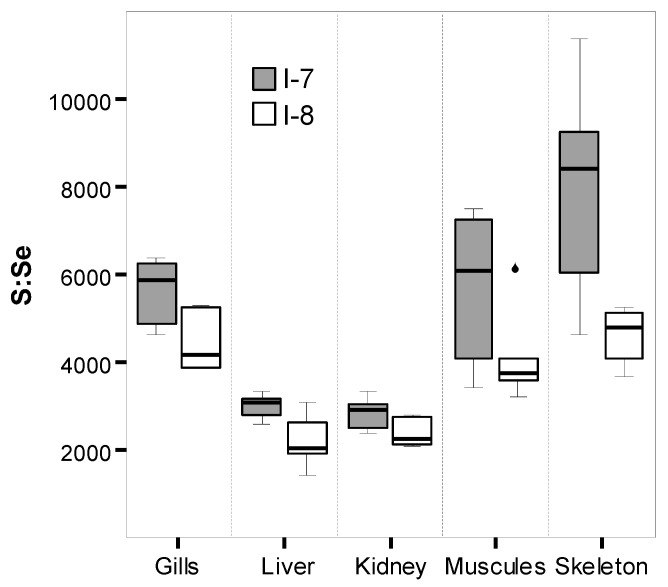
Molar relations of sulfur to selenium (S:Se) in whitefish organs and tissues from the thermal pollution area (I-7) and the natural habitat area (I-8).

As opposed to terrestrial animals, the evolutionary increased reliance on Se is evident in the larger selenoproteomes of aquatic animals, including fish, which have the largest number of selenoproteins among all biota [28]. Unlike the majority of essential trace elements, Se is not coordinated with proteins, but is covalently incorporated as a selenocysteine (SeCys) residue. Groups of selenoenzymes are glutathione peroxidases (whose function is to reduce hydroperoxides to corresponding alcohols at the expense of glutathione), thioredoxin reductases (that maintain cellular redox status by maintaining reduced cysteine), iodothyronine deiodinases (which activate the prohormone thyroxine (T_4_) to the active thyroid hormone triiodothyronine (T_3_) and catalyze the inactivation of T_4_ to reverse T_3_ (rT_3_) and T_3_ to diiodothyronine (T_2_)), and selenophosphate synthetases (which synthesizes selenoproteins by producing the active (phosphorylated) form of Se) [29]. In contrast to other amino acids, SeCys is not reused in subsequent cycles of protein synthesis but must be degraded to release inorganic Se for the synthesis of SeCys, which is placed in the active sites of selenoenzymes [30]. The scheme of Se metabolism is shown in Figure 3. If Se is specifically incorporated into essential selenoproteins as SeCys, then in all other proteins, selenomethionine (SeMet) replaces methionine in an unregulated and dose-dependent manner, causing Se toxicity, although SeMet can be considered an unregulated pool of Se for eventual SeCys synthesis at a normal dietary uptake [29]. An adequate amount of dietary Se is essential for the proper body growth of fish [31], and an increase in the growth rate is observed under warmer conditions [32]. Conversely, fish growth rates and lipid contents were significantly decreased with a high SeMet diet [33]. The dose-dependent substitution of S for Se in methionine, as a consequence of dietary Se exposure, was illustrated in an experiment with a cage study using wild lake chub (*Couesius plumbeus*), collected from a reference lake and lake receiving waters of a uranium-processing mill in Northern Saskatchewan, Canada [34]. A decreasing whole-body S isotope signature and an increasing proportion of SeMet-like compounds (determined by X-ray absorption spectroscopy) were observed for the Se-spiked diet treatment groups after 21 days. Since Se has a high potential to biomagnify in aquatic food webs [29], it can be assumed that whitefish receive Se with food slightly more in the warmer water of area I-7. However, having examined the molar ratios of sulfur to Se, these ratios are significantly higher in all organs and tissues (excluding the muscles) of the whitefish from area I-7, compared to the whitefish from area I-8 (Figure 4). It can be assumed that Se is largely included in the essential SeCys in the whitefish from area I-7, while SeMet pool is formed in the whitefish from area I-8. In many cases, S is replaced by Se in methionine, and methionine is replaced by SeMet. On the one hand, the whitefish in the thermal influence area are not exposed to any toxic effects of Se; on the other hand, they are the most dependent on its deficit. The largest decrease in Se content (more than 1.5 times) is observed in the liver (the dominant site of selenoprotein synthesis and catabolism) of the whitefish from area I-7 (Table 1), especially with an increase in FCF (Table 3).

The decrease in Mo bioaccumulation in the gills, kidney, muscles and skeleton with an increasing FCF (Table 3) is apparently associated with an intensive metabolism and, in particular, sulfur-containing amino acids. Molybdenum is a cofactor of at least seven enzymes, the principal of which are xanthine oxidase/dehydrogenase (that is involved in the oxidation of purine and pyrimidines, as well as other nitrogen-containing heterocyclic compounds) and sulfite oxidase (that is found in the mitochondrial intermembrane space, oxidizes sulfite to sulfate, and is the terminal step in the metabolism of sulfur-containing amino acids) [36]. Tungsten metabolism is related to that of Mo, which it closely resembles in its chemical properties [37]. Molybdate [36], like selenate [29] uses a sulfate transport system. First, a 3Na^+^/SO_4_^2^^−^–co-transporter (SLC13s1) moves both Na^+^ and SO_4_^2^^−^ across the apical membrane, driven by the Na^+^ gradient produced by NKA on the basolateral membrane; then, the SO_4_^2^^−^ is moved across the basolateral membrane by an SO_4_^2^^−^/2HCO_3_^−^–exchanger (SLC26a1), which is energized by an SO_4_^2^^−^ gradient across the basolateral membrane [18]. Evidently, Si also uses the sulfate transport system. An enhanced excretion of Na by kidneys of the whitefish from the thermal affected area probably contributes to the Na-dependent transport of Se, Mo, W, and Si (Table 1 and Table 2). Se, Mo, Si, excretion increases with an increasing FCF (Table 3). Since Ni also uses Mg transporters [18], the renal excretion of Mg seems to favor Ni excretion, especially with an increase in FCF (Table 3).

Selenium interacts with a variety of other trace elements, primarily in an antagonistic fashion [29]. One of the unfavorable interactions of Se is associated with an increase in Hg bioaccumulation in the liver of fish (Table 2). A vivid antagonistic example was observed under conditions of strong pollution from a copper smelter, and a sufficient accumulation of Se significantly reduced Hg bioaccumulation in the organs and tissues of bream (*Abramis brama* L.), compared to those under background conditions [38]. On the other hand, an increase in Hg bioaccumulation in the liver and muscles of fish was observed along a strong temperature gradient from the water bodies of subarctic regions to the water bodies of Southern Russia [39]. Mercury biomagnifies to higher trophic levels in food webs and methylmercury (MeHg), accounting for 91% of the mercury found in the whitefish (*Coregonus clupeaformis*) from Northern Lake Huron [40]. In Fennoscandian, an increase in the muscle Hg concentration in 6 fish species and Hg bioaccumulation rate in whitefish (*Coregonus lavaretus*) was observed, according to the temperature gradient from the cold pristine oligotrophic lakes in the north to the warmer and increasingly human-altered mesotrophic and eutrophic systems in the south, where, apparently, mercury methylation is more intensive [41]. Methylmercury is absorbed effectively (>90%) from the gut and enters tissues as a cysteine-bound conjugate that mimics the amino acid methionine, moving freely into cells via amino acid transport proteins [7]. Methylmercury is by definition a highly specific irreversible selenoenzyme inhibitor, and intracellular MeHg tends to diminish the amount of Se that is biologically available for normal selenoenzyme synthesis, especially as Hg:Se molar ratios approach a 1:1 stoichiometry [35]. The molar ratio of Hg:Se averaged 0.007 (maximum—0.012) in the liver of the whitefish from area I-7, while this ratio was 0.004 (maximum—0.005) for those from area I-8. These ratios are still small.

Another unfavorable interaction of Se is associated with the As accumulation, which is significant in all organs and tissues (excluding muscles) in the whitefish from the thermal affected area, whereas As does not accumulate, and its concentrations were below the detection threshold under natural conditions (Table 2). Both arsenite (As [III]) and arsenate (As [V]) interact antagonistically with Se, and the interaction involves complexation with endogenous thiols [29]. However, the gene-encoded MRP2 is known to transport a wide range of anionic substrates, including As–glutathione conjugates [42]. The inactivation of the selenoenzymes may be increased in the liver of the whitefish from area I-7. The molar ratio (Hg + As): Se averaged 0.049 (maximum 0.232) in the liver of the whitefish from area I-7, while this ratio remains the same as Hg:Se for the whitefish from area I-8.

Interactions between Cu and Se in fish decrease the tissue-specific Se accumulation, and the elevated dietary Cu reduced the concentrations of Se in the liver [29]. Thus, Cu not only lowers the energy potential of the liver, as shown above, but it also reduces the Se accumulation in the liver, and their accumulation is negatively correlated (r = −0.606, *p* < 0.05). Moreover, the Cu accumulation increases with an increasing FCF (Table 3). For example, the upper threshold for the Cu concentration is specified for the yellow perch (*Perca flavescens*) liver at 38.8 μg/g dry weight, with higher values representing a risk of toxicity [43]. The concentrations of Cu exceeded this threshold in the liver of all whitefish under conditions of warm water exposure (Table 1).

A reduced accumulation of Si was found in the gills and kidneys, but the increased accumulation in the liver of whitefish from the thermal affected area (Table 1) may indicate support for liver function. Moreover, the Si accumulation decreases in the kidney but increases in the liver with an increasing FCF (Table 3). In humans, the highest concentrations of silica occur in connective and elastic tissues and especially the aorta, where it appears to function as a crosslinking agent that stabilizes collagen and presumably strengthens the vasculature [44].

It is worth paying attention to the fact that, being inert in its chemical and biological properties, Zr accumulates more in the liver, muscles and skeleton of the whitefish under conditions of warmer waters (Table 2). Moreover, the Zr accumulation is enhanced in the liver and skeleton with an increasing blood hemoglobin concentration and FCF (Table 3).

## 5. Conclusions

Whitefish living in the thermal pollution area of subarctic Lake Imandra differ favorably from whitefish in the natural temperature environment in terms of their physiological state. Firstly, the hemoglobin concentrations in the fish blood were in the range of the environmental optimum, and secondly, they had a higher somatic growth, estimated by Fulton’s condition factor. In general, a decreased bioaccumulation of most essential and non-essential metals, metalloids, and trace metals was observed, especially in the gills and kidney of the whitefish under thermal exposure. A positive effect was the better elimination of Cd, Ni, Al, W, and Sb.

One of the main adaptive mechanisms of ion regulation is the intense metabolism of Na due to the high respiratory activity of the whitefish in warmer water. The increased accumulation of Rb in all organs and tissues of the whitefish under thermal impact is associated with an intense metabolism of Na, namely, the interconnection of Na and K transport. An unfavorable effect of the high renal excretion of Na is the Na-dependent transport of sulfates, which apparently stimulates the excretion of Se, Mo, and Si.

Under the condition of an increased water temperature, the main metabolic fish need is Se in the absence of environmental pollution with it. On the one hand, the whitefish in the thermal influence area are not exposed to any toxic effects of Se; on the other hand, they are most dependent on its deficit, which was most evident in the liver of the fish. In general, the liver is the target organ of thermal impact. A high permeation of Cu, especially in the ultra-fresh soft water of the lake, and its overaccumulation in the liver, as well as a loss of Mg, contribute to a reduced energy potential of the liver. Adaptation to thermal effects may reduce the ability of the whitefish population to adapt to other stress factors. Due to the intense metabolism of selenoproteins, the risks of toxic effects and the bioaccumulation of Hg, As, and Cu are likely to arise in the case of an increased existing stressor or the appearance of new ones.

## Figures and Tables

**Table 1:** Average values and standard errors of the essential and conditionally essential metal concentrations in the water, organs and tissues of the whitefish caught from area I-7 (top line) and area I-8 (bottom line). The maximum values are shown in brackets. The bold type shows a reliable difference (with a significance level *p* < 0.05) in the average values of the metal concentrations.

Metal	Water	Gills	Liver	Kidney	Muscles	Skeleton
	μg/L	μg/g Dry Weight				
Ca	3740	30,991 ± 2232	654 ± 180	880 ± 147	2369 ± 891	**155,291** ± 1500
	3590	31,909 ± 1840	627 ± 161	1318 ± 283	1006 ± 310	**148,031** ± 3047
Mg	1120	1083 ± 73	**713** ± 73	**883** ± 91	1367 ± 76	2033 ± 30
	1090	1163 ± 34	**878** ± 37	**1307** ± 131	1233 ± 65	2120 ± 37
Na	7270	6418 ± 449	**3558** ± 387	**6536** ± 556	815 ± 96	**7055** ± 163
	6250	5745 ± 347	**5316** ± 431	**8979** ± 469	834 ± 59	**6577** ± 106
K	1470	9941 ± 458	10,276 ± 562	10,583 ± 1235	18,264 ± 1111	**3086** ± 220
	1300	10,080 ± 534	12,105 ± 835	12,088 ± 826	17,646 ± 1216	**3850** ± 188
S	5300	10,857 ± 340	10,684 ± 621	10,924 ± 912	**10,479** ± 85	4613 ± 140
	4245	10,712 ± 225	11,946 ± 390	11,096 ± 514	**9679** ± 339	4632 ± 220
P	0.8	26,150 ± 1601	**14,099** ± 985	14,031 ± 1248	12,724 ± 964	**83,475** ± 960
	0.8	26,587 ± 660	**17,878** ± 679	16,413 ± 514	11,675 ± 865	**78,618** ± 1495
Fe	6.1	**168** ± 14	155 ± 19	289 ± 39	13.9 ± 2.6	13.3 ± 4.3
	13.9	**385** ± 57	172 ± 23	310 ± 24	13.0 ± 2.8	12.6 ± 3.3
Zn	0.44	504 ± 122	290 ± 82	231 ± 29	19.3 ± 1.3	120 ± 12
	2.65	713 ± 113	334 ± 78	274 ± 43	17.6 ± 1.3	141 ± 16
Cu	2.3	3.0 ± 0.4	**68.1** ± 11.3	27.6 ± 16.5	1.2 ± 0.2	0.7 ± 0.2
	2.8	2.2 ± 0.1	**32.9** ± 7.4	10.2 ± 0.9	1.3 ± 0.1	0.6 ± 0.2
Mn	1.3	19.6 ± 4.4	**7.21** ± 0.53	3.98 ± 0.82	2.23 ± 1.32	49.7 ± 13.8
	1.4	25.0 ± 5.3	**8.62** ± 0.38	4.27 ± 0.40	0.84 ± 0.07	36.9 ± 3.8
Co	<0.1	**<0.04** (0.10)	0.10 ± 0.02	1.09 ± 0.22	<0.04	<0.04
	<0.1	**0.13** ± 0.03	0.12 ± 0.02	0.74 ± 0.16	<0.04	<0.04
Se	<0.3	**4.8** ± 0.4	**8.7** ± 0.3	**9.4** ± 0.7	4.9 ± 0.7	**1.6** ± 0.3
	<0.3	**6.0** ± 0.4	**14.2** ± 1.8	**11.6** ± 0.7	6.1 ± 0.5	**2.5** ± 0.2
Mo	1.03	**0.11** ± 0.02	0.67 ± 0.07	**0.59** ± 0.08	0.02 ± 0.02	**0.18** ± 0.03
	0.74	**0.21** ± 0.03	0.73 ± 0.07	**0.94** ± 0.13	0.02 ± 0.01	**0.34** ± 0.07
B	<8	**<0.3** (3.7)	**<0.3**	1.0 ± 0.4	<0.3 (0.5)	**<0.3**
	<8	**2.2** ± 0.4	**1.0** ± 0.4	1.6 ± 0.5	<0.3	**2.4** ± 0.7
Br	12.3	65.5 ± 18.7	46.0 ± 7.1	**50.8** ± 5.7	38.0 ± 8.6	**32.8** ± 16.1
	8.8	72.9 ± 13.3	54.2 ± 7.5	**110** ± 22.6	37.6 ± 5.9	**80.2** ± 15.1
Cr	<0.5	**<0.3** (3.7)	<0.3	<0.3	<0.3	<0.3 (0.6)
	<0.5	**0.9** ± 0.1	<0.3	<0.3	<0.3	<0.3 (0.7)
Ni	1.5	1.1 ± 0.1	0.6 ± 0.2	5.4 ± 1.3	0.4 ± 0.2	**<0.1**
	1.3	1.5 ± 0.4	1.1 ± 0.3	7.6 ± 1.5	0.1 ± 0.1	**0.2** ± 0.1
Si	1.27	**54.8** ± 20.5	**18.5** ± 1.1	**40.7** ± 7.6	18.4 ± 1.2	12.5 ± 1.2
	1.38	**272** ± 38.4	**13.1** ± 0.8	**68.9** ± 7.2	15.0 ± 3.2	13.1 ± 4.6
Li	0.54	**0.017** ± 0.005	0.002 ± 0.002	0.006 ± 0.003	<0.003 (0.008)	0.026 ± 0.003
	0.58	**0.095** ± 0.016	0.003 ± 0.001	0.016 ± 0.003	<0.003	0.030 ± 0.005

**Table 2:** Average values and standard errors of the toxic metal concentrations in the water, organs and tissues of the whitefish caught from area I-7 (top line) and area I-8 (bottom line). The maximum values are shown in brackets. The bold type shows a reliable difference (with a significance level *p* < 0.05) in average values.

Metal	Water	Gills	Liver	Kidney	Muscles	Skeleton
	μg/L	μg/g Dry Weight				
Hg	<0.01	0.064 ± 0.006	**0.162** ± 0.021	0.200 ± 0.022	0.098 ± 0.021	**0.018** ± 0.006
	<0.01	0.069 ± 0.009	**0.120** ± 0.007	0.193 ± 0.025	0.099 ± 0.025	**0.033** ± 0.003
Tl	0.001	0.042 ± 0.003	0.473 ± 0.054	0.231 ± 0.111	0.031 ± 0.004	0.020 ± 0.002
	0.002	0.045 ± 0.006	0.564 ± 0.119	0.100 ± 0.025	0.032 ± 0.006	0.025 ± 0.004
Be	<0.006	**<0.002**	<0.002	0.023 ± 0.023	<0.002	<0.002
	<0.006	**0.004** ± 0.001	<0.002	<0.002	<0.002	<0.002
Cd	<0.004	0.076 ± 0.014	0.172 ± 0.034	**2.01** ± 0.49	<0.004	0.013 ± 0.003
	<0.004	0.057 ± 0.007	0.250 ± 0.041	**3.46** ± 0.35	<0.004	0.008 ± 0.002
Pb	1.43	0.24 ± 0.10	0.05 ± 0.02	0.07 ± 0.03	0.01 ± 0.01	0.06 ± 0.01
	0.2	0.27 ± 0.04	0.05 ± 0.01	0.07 ± 0.01	0.01 ± 0.004	0.10 ± 0.02
Ag	<0.005	0.003 ± 0.001	0.100 ± 0.024	0.039 ± 0.029	<0.002	<0.002
	<0.005	0.012 ± 0.007	0.068 ± 0.011	0.013 ± 0.004	0.002 ± 0.002	0.016 ± 0.011
W	0.044	0.034 ± 0.006	**0.003** ± 0.001	**0.040** ± 0.012	0.002 ± 0.001	0.158 ± 0.029
	0.031	0.040 ± 0.009	**0.007** ± 0.001	**0.077** ± 0.012	<0.002	0.155 ± 0.029
V	0.13	<0.2 (0.4)	<0.2	0.3 ± 0.2	<0.2	0.3 ± 0.2
	0.09	0.7 ± 0.3	0.2 ± 0.1	0.6 ± 0.2	<0.2	0.4 ± 0.4
Te	<0.006	**<0.004**	0.012 ± 0.003	0.031 ± 0.007	<0.004	<0.004
	<0.006	**0.008** ± 0.001	0.016 ± 0.002	0.034 ± 0.005	<0.004	<0.004
Sb	0.042	**<0.002**	**<0.002**	**<0.002**	<0.002	0.016 ± 0.006
	0.037	**0.004** ± 0.0004	**0.003** ± 0.001	**0.005** ± 0.0005	<0.002 (0.007)	0.017 ± 0.007
Bi	<0.004	0.0021 ± 0.0005	0.0022 ± 0.0010	0.0109 ± 0.0082	0.0005 ± 0.0002	0.0009 ± 0.0003
	<0.004	0.0026 ± 0.0004	0.0012 ± 0.0002	0.0037 ± 0.0006	0.0010 ± 0.0002	0.0017 ± 0.0006
As	<0.007	0.15 ± 0.09	0.31 ± 0.26	**0.21** ± 0.11	<0.04	**0.21** ± 0.13
	<0.007	<0.04	<0.04	**<0.04**	<0.04	**<0.04**
Sr	44.2	225 ± 23	5.1 ± 1.4	5.9 ± 0.9	16.5 ± 6.5	**1083** ± 127
	36.7	182 ± 11	5.2 ± 1.7	9.2 ± 2.1	4.1 ± 1.5	**812** ± 46
Al	7.6	**16.5** ± 7.9	**7.3** ± 1.3	**10.8** ± 2.3	1.7 ± 1.3	5.0 ± 0.7
	12.3	**195** ± 36	**14.2** ± 3.2	**22.0** ± 3.7	7.0 ± 4.7	10.9 ± 3.2
Ti	<0.5	**1.3** ± 1.0	<0.5 (1.2)	<0.5 (2.0)	<0.5	1.7 ± 0.4
	<0.5	**16.7** ± 2.8	<0.5	0.5 ± 0.5	<0.5	3.0±0.9
Zr	0.004	0.10 ± 0.04	**0.08** ± 0.02	0.12 ± 0.04	**0.04** ± 0.01	**0.06** ± 0.01
	0.012	0.08 ± 0.02	**0.02** ± 0.01	0.08 ± 0.01	**<0.02**	**0.02** ± 0.01
Rb	2.03	**35.0** ± 1.7	**41.0** ± 3.7	**40.4** ± 4.8	**50.3** ± 3.3	**9.5** ± 0.7
	1.66	**23.0** ± 2.0	**31.6** ± 2.2	**27.1** ± 2.0	**31.1** ± 4.1	**7.5** ± 0.5
Sn	<0.012	<0.005	0.005 ± 0.005	<0.005	<0.005	**<0.005**
	0.045	0.022 ± 0.017	0.008 ± 0.007	0.014 ± 0.007	<0.005 (0.017)	**0.019** ± 0.007
Ba	4.47	**3.16** ± 0.51	0.47 ± 0.09	**0.90** ± 1.86	0.37 ± 0.12	8.17 ± 0.93
	6.13	**5.37** ± 0.94	0.41 ± 0.08	**1.55** ± 0.22	0.43 ± 0.12	11.4 ± 1.62
U	0.036	**0.017** ± 0.002	0.006 ± 0.001	0.028 ± 0.010	0.007 ± 0.003	**0.034** ± 0.004
	0.039	**0.028** ± 0.004	0.009 ± 0.002	0.029 ± 0.006	0.004 ± 0.002	**0.064** ± 0.011
Cs	0.012	0.156 ± 0.009	0.150 ± 0.028	0.232 ± 0.038	**0.231** ± 0.021	0.059 ± 0.010
	0.046	0.174 ± 0.026	0.123 ± 0.034	0.304 ± 0.115	**0.139** ± 0.024	0.042 ± 0.004

**Table 3:** Correlation coefficients of the metal concentrations in the organs and tissues of whitefish, depending on the blood hemoglobin concentrations (Hb) and Fulton’s condition factor (FCF).

Element	Gills	Liver	Kidney	Muscles	Skeleton
**Hb**					
Na	–	–	−0.683 **	–	–
P	–	−0.590 *	–	–	–
Cu	–	–	0.627 *	–	–
Tl	–	–	0.600 *	–	–
Te	−0.649 *	–	–	–	–
Sb	−0.630 *	−0.627 *	−0.659 **	–	–
Sr	0.743 ***	–	–	–	0.838 ****
Zr	–	0.655 **	–	–	0.703 ***
Rb	0.759 ***	–	0.675 **	0.776 ***	–
FCF					
Na	–	–	−0.676 **	–	–
Fe	−0.635 *	–	–	–	–
Cu	–	0.640 *	–	–	–
Co	−0.681 **	–	–	–	–
Se	–	−0.738 ***	−0.596 *	–	–
Mo	−0.683 **	–	−0.635 *	−0.602 *	−0.607 *
B	−0.674 **	–	–	–	−0.692 **
Cr	−0.659 *	–	–	–	–
Ni	–	–	−0.575 *	–	−0.905 ****
Si	–	0.638 *	−0.659 **	–	–
Li	−0.694 **	–	–	–	–
Be	−0.637 *	–	–	–	–
Ag	−0.669 **	–	–	–	−0.697 **
W	–	−0.626 *	–	–	–
Sb	−0.627 *	−0.641 *	–	–	–
Al	−0.651 **	–	–	–	–
Ti	−0.603 *	–	–	–	–
Zr	–	0.619 *	–	–	0.728 ***
Rb	−0.600 *	–	–	–	–

Note. The significance level is indicated as follows: * *p* < 0.05, ** *p* < 0.01, *** *p* < 0.005, **** *p* < 0.001. A dash means that either there is no correlation, or it is insignificant.
